# Reduction in antioxidant enzyme expression and sustained inflammation enhance tissue damage in the subacute phase of spinal cord contusive injury

**DOI:** 10.1186/1423-0127-18-13

**Published:** 2011-02-07

**Authors:** Chih-Yen Wang, Jen-Kun Chen, Yi-Ting Wu, May-Jywan Tsai, Song-Kun Shyue, Chung-Shi Yang, Shun-Fen Tzeng

**Affiliations:** 1 Department of Life Sciences, National Cheng Kung University, Tainan, Taiwan; 2 Center for Nanomedicine Research, National Health Research Institutes, Zhunan, Taiwan; 3 Institute of Biomedical Sciences, Academia Sinica, Taipei, Taiwan; 4 Graduate Institute of Biomedicine and Biomedical Technology, National Chi-Nan University, Puli, Taiwan

## Abstract

**Background:**

Traumatic spinal cord injury (SCI) forms a disadvantageous microenvironment for tissue repair at the lesion site. To consider an appropriate time window for giving a promising therapeutic treatment for subacute and chronic SCI, global changes of proteins in the injured center at the longer survival time points after SCI remains to be elucidated.

**Methods:**

Through two-dimensional electrophoresis (2DE)-based proteome analysis and western blotting, we examined the differential expression of the soluble proteins isolated from the lesion center (LC) at day 1 (acute) and day 14 (subacute) after a severe contusive injury to the thoracic spinal cord at segment 10. In situ apoptotic analysis was used to examine cell apoptosis in injured spinal cord after adenoviral gene transfer of antioxidant enzymes. In addition, administration of chondroitinase ABC (chABC) was performed to analyze hindlimb locomotor recovery in rats with SCI using Basso, Beattie and Bresnahan (BBB) locomotor rating scale.

**Results:**

Our results showed a decline in catalase (CAT) and Mn-superoxide dismutase (MnSOD) found at day 14 after SCI. Accordingly, gene transfer of SOD was introduced in the injured spinal cord and found to attenuate cell apoptosis. Galectin-3, β-actin, actin regulatory protein (CAPG), and F-actin-capping protein subunit β (CAPZB) at day 14 were increased when compared to that detected at day 1 after SCI or in sham-operated control. Indeed, the accumulation of β-actin^+ ^immune cells was observed in the LC at day 14 post SCI, while most of reactive astrocytes were surrounding the lesion center. In addition, chondroitin sulfate proteoglycans (CSPG)-related proteins with 40-kDa was detected in the LC at day 3-14 post SCI. Delayed treatment with chondroitinase ABC (chABC) at day 3 post SCI improved the hindlimb locomotion in SCI rats.

**Conclusions:**

Our findings demonstrate that the differential expression in proteins related to signal transduction, oxidoreduction and stress contribute to extensive inflammation, causing time-dependent spread of tissue damage after severe SCI. The interventions by supplement of anti-oxidant enzymes right after SCI or delayed administration with chABC can facilitate spinal neural cell survival and tissue repair.

## Background

Traumatic spinal cord injury (SCI) causes permanent paralysis in patients due to low regeneration of the CNS [1]. The events occurring immediately after SCI include neuronal fiber damage, mass ischemic neural cell necrosis and apoptosis, metabolic disturbances, the destruction of microvasculature, inflammation, lipid peroxidation, free radical production, demyelination, and glial scar formation, leading to extensive secondary tissue injuries [1-3]. Robust cell death in the injured region happens from seconds to weeks after SCI, which results in the formation of the cavities or cysts that blockades the ascending and descending neurotransmission [2,4]. In response to local inflammation after SCI, microglia, CNS-resident macrophages, are activated, which trigger inflammatory reaction in the injured center [2,5]. Accordingly, it is thought that the inflammatory reactions could take place over weeks after SCI, which induce the recruitment of neutrophils, macrophages and T cells from hours to weeks after injury [2,5-7]. In addition, astrocytes become reactivated with an increase in number and hypertrophy, a process so called as gliosis. The event forms glial scar to prevent the spread of injury factors and to inhibit the expansion of inflammatory reactions [1,8]. Although the degenerative axon of the uninjured cell body can be stimulated to be regenerative, the scar structure is extremely compact which creates a physical barrier to axon regeneration. Moreover, the scar tissue contains the inhibitors to axon outgrowth, producing a microenvironment that is not beneficial for tissue repair after SCI [1,9,10].

Recently, DNA microarray and proteome analysis have been used to understand SCI-induced pathophysiology and to find potential therapeutic targets. Several studies using genechip microarray have described gene expression changes from impact to months after SCI. By using the technology, genes associated with transcription and inflammation have been found to be upregulated at the early stage (from minutes to weeks) after SCI, while the genes of structural proteins and genes encoding proteins involved in neurotransmission are downregulated [2,11,12]. Although an increased expression of growth factors, axonal guidance factors, extracellular matrix molecules and angiogenic factors can be observed in the chronic phase (days to years) following SCI, oxidative stress-related genes and proteases are still increased [2,13,14]. The proteomic profile has also shown that several proteins involved in neural function, cell adhesion/migration, stress/metabolism, and apoptosis were detected at day 1 post SCI [15]. Recent proteome-based studies have also reported dynamic protein change profile in the injured spinal cord which were collected from 2 cm length of the cord segment at 8 hour, day 1, day 3 and day 5 after moderate contusive injury [16]. A subacute time point (approximately 2 weeks) has been suggested to be an appropriate time window for treatment since it could be more favorable for axon regeneration and behavioral recovery than that carried out at the acute stage of SCI [17]. However, global changes of proteins in the injured epicenter at the subacute stage of SCI remain to be elucidated.

Since a contusive injury to the spinal cord is most similar to crush and fracture spinal cord injuries in human [18], a well-characterized NYU impactor device was used to induce severe spinal cord contusion. Through proteomics-based analysis, the study was aimed at examining differential protein expression in the lesion center (LC) of the injured spinal cord isolated from rats at day 14 (subacute SCI) or from rats at day 1 (acute SCI) post SCI. Western blot analysis and immunofluorescence were also conducted to validate the proteome analysis by examining the expression profile of proteins identified in the LC at the different survival time points after SCI. Our results provide target molecules for the potential treatments which can efficiently improve neural survival in the injured spinal cord and to enhance hindlimb recovery in rats with SCI.

## Materials and methods

### Spinal cord injury

Female adult Sprague-Dawley rats (250 g ± 30; n = 45 rats) were anesthetized, and their spinal cords were exposed by laminectomy at the level of T9/T10. A 10-g rod was dropped onto the laminectomized cord from a height of 50 mm (severe) using a device developed at the New York University [19,20]. During surgery the rectal temperature was maintained at 37°C using a thermostatically regulated heating pad and bladder evacuation was then applied daily. Antibiotics (sodium ampicillin 80 mg/kg) were injected post surgery. Animal care was provided in accordance with the Laboratory Animal Welfare Act and Guide for the Care and Use of Laboratory Animals approved by Institutional Animal Care and Use Committee of National Cheng Kung University.

### Sample preparation for 2-DE

The spinal segments (4-5 mm) containing the LC were isolated at day 1 and 14 post severe SCI (n = 10 rats). The samples isolated from the injured spinal cord at the two time points (day 1 and 14) were prepared in parallel for 2-DE. In brief, the tissues were homogenized in 0.2 ml of cold detergent free lysis buffer consisting of 40 mM Tris, 40 mM sodium acetate and protease inhibitor cocktail for 30 min, followed by sonication. The homogenate was centrifuged at 10,000 g for 30 min at 4°C to remove insoluble debris. The proteins were then precipitated by cold acetone with 10% trichloroacetic acid overnight. After centrifugation, the protein pellet was washed with cold acetone followed by air drying, and then resuspended in the rehydration buffer containing 8 M urea, 4% CHAPS, 0.2% Bio-Lyte 3/10 (Bio-Rad, Hercules, CA) and 50 mM dithiothreitol (DTT) (Sigma, St. Louis, MO). Protein concentration was assessed using a Bio-Rad detergent compatible kit.

### 2-DE

For the first-dimension IEF, pH 3-10 non-linear range IPG strips (11 cm) were rehydrated with 200 μl of solubilized sample (200 μg protein amount) for 12 h before the sample was separated by IEF at 100 V for 0.5 h, 500 V for 0.5 h, 1000 V for 1 h, 5000 V for 1 h, and finally 8000 V for 3 h. Prior to the second dimension SDS-PAGE, the IPG strips were equilibrated with 2 ml of equilibration buffer consisting of 0.375 M Tris, 6 M urea, 2% SDS, 20% glycerol and 0.02 g/ml DTT at 25°C for 15 min followed by equilibration in 0.375 M Tris, 6 M urea, 2% SDS, 20% glycerol and 0.025 g/ml iodoacetamide (IAA) at 25°C for 15 min. The second dimensional SDS-PAGE used a 10% separating gel and was performed without a stacking gel. The equilibrated IPG gel strip was placed on top of the SDS-PAGE gel and was sealed with 0.5% low-melting temperature agarose with 0.01% bromophenol blue. Electrophoresis was carried out at 180 V until the tracking dye reached the bottom of the gel. The gel was subjected to silver staining according to the method described by Tsai et al. [21].

### Quantitative analysis of the proteins in the 2-DE

Protein pattern images in 2-DE SDS-PAGE were obtained using a high-resolution scanner and the amount of protein in each spot was estimated using ImageMaster 2D Platnum software (v7.0, GE Healthcare Bio-Sciences AB, Uppsala, Sweden). The volume of a protein spot was defined as the sum of the intensities of the pixel units within the protein spot. To correct quantitative variations in the intensity of protein spots, spot volumes were normalized as a percentage of the total volume of all the spots present in each gel.

### Protein identification by mass spectrometer

The protein spots were manually excised from silver stained 2-DE gels, destained, washed and in-gel digested as follows. The gel pieces were transferred to the destain solution (0.1 g K_3_Fe(CN)_6 _and 0.16 g Na_2_S_2_O_3 _solved in 10 ml double deionized water) for another 10 minutes, reduced with 50 mM DTT in 25 mM ammonium bicarbonate (pH 8.5) at 37°C for one hour, and then alkylated with 100 mM IAA in 25 mM ammonium bicarbonate (pH 8.5) at 37°C for one hour. After the gel pieces were dehydrated and dried by SpeedVac concentrator, the dried gel pieces were rehydrated with 20 ng of modified trypsin (sequencing grade, Promega, Madison, WI, USA) in 25 mM ammonium bicarbonate (pH 8.5) at 37°C for 16 h. The tryptic peptide mixture was concentrated and immediately redissolved for protein identification. Matrix assisted laser desorption ionization time-of-flight mass spectrometer (MALDI-TOF MS) (Autoflex III, Bruker Daltonics, Bremen, Germany) was employed for peptide mass fingerprinting (PMF) analysis. The MALDI-TOF MS operated with reflectron mode was externally calibrated with peptide calibration standard I (Bruker Daltonics) for each batch of samples and neighboring calibration with angiotensin II (1046.5418 *m/z*), [Glu]-fibrinopeptide B (1570.6774 *m/z*), and ACTH fragment 18-39 (2465.1983 *m/z*) for each sample to achieve 50 ppm or better of mass measurement accuracy in the range of 920-3500 *m/z*. The mass spectra were acquired by flexControl software (v3.0, Bruker Daltonics) and processed by flexAnalysis software (v3.0, Bruker Daltonics). To generate peak lists from raw MS data, the sophisticated number assigned program (SNAP) peak detection algorithm was used, filtered with S/N >3, and then smoothed with SavitzkyGolay algorithm for 0.15 *m/z *peak width and 4 cycles. We subsequently searched all peak lists against Mascot engine with Swiss-Prot database (Release version 56.6 of 16-Dec-2008). The search parameters allowed for one missed cleavage tryptic peptides, oxidation of methionine, carbamidomethylation of cysteine and at least 50 ppm mass accuracy. The probability-based Mowse scores with the *p *value less than 0.05 were accepted for protein identification.

### Western blotting

The protein extracts (30 μg/lane) used for 2-DE were separated on 10% SDS-PAGE and then transferred to a nitrocellulose membrane (Millipore, Billerica, MA). The membrane was then probed overnight at 4°C with primary antibodies at the appropriate dilution, and then incubated with HRP-conjugated secondary antibodies (Jackson ImmunoResearch Laboratories, West Grove, PA, USA) for 1 h at room temperature. The detection was carried out by using ECL chemilluniscence (Amersham Pharmacia, Buckinghamshire, United Kingdom). The antibodies used for this study are listed as follows: anti-β-actin, anti-actin regulatory protein (CAPG) and anti-cathepsin D (CATD) antibodies (Santa Cruz Biotechnology, Santa Cruz, CA); anti-GFAP and anti-GAPDH antibodies (Chemicon, Temecula, CA); anti-superoxide dismutase [Mn] (MnSOD) antibody (Stressgen, Ann Arbor, MI); anti-dihydropyrimidinase-related protein-2 (DPYL-2)/CRMP-2, DPYL-5, catalase (CAT), heat shock protein-60 (Hsp60), Hsp27, galectin-3 (LEG3), latexin (LXN), peroxiredoxin-1 (Prx1), and Prx6 antibodies (ABcam, Cambridge, MA); anti-extracellular signal-regulated kinase (ERK) antibody (Cell Signaling, Beverly, MA, USA);F-actin-capping protein subunit β (CAPZB) antibody (Everest biotech, UK); anti-Iba1 antibody (Wako Pure Chemical, Osaka, Japan).

### Analysis of CSPG in the injured spinal cord tissues

Spinal tissue blocks (approximately 4-5 mm thickness/block) were collected from the LC and from rostral or caudal regions adjacent to the epicenter at the different survival time points after severe SCI. The tissues were homogenized in extraction solution containing 40 mM Tris, 40 mM sodium acetate and protease inhibitor cocktail (Sigma) using the sonicator. Protein concentration was assayed using the Bio-Rad DC kit (Bio-Rad, Hercules, CA). Protein extracts (30 μg) were digested at 37°C for 3-5 h with 0.03 U of chondroitinase ABC (chABC; Sigma), loaded onto 10% SDS-PAGE, and then transferred to nitrocellulose membrane. The membrane was incubated with anti-chondroitin-4-sulfate antibody (Chemicon, Temecula, CA) overnight at 4°C and HRP-conjugated secondary antibody for 1 h at room temperature. The detection was carried out by using ECL chemilluniscence.

### Immunohistochemistry

Animals were perfused intracardially with 0.9% cold NaCl, followed by 4% paraformaldehyde in 0.1 M phosphate buffer. The spinal cords were removed, postfixed in 4% paraformaldehyde overnight, and then cryoprotected in PBS containing 30% (w/v) sucrose for 3 days. The cord (approximately 2 cm in length covering the epicenter) was excised, embedded in Tissue Tek OCT (Sakura Finetek, CA), and then longitudinally sectioned at 20 μm thickness. Tissue sections were collected onto glass slides and dried at 37°C. The tissue sections were incubated with anti-β-actin, anti-GFAP (Chemicon), anti-CD11b (BD Biosciences, San Jose, CA, USA), and anti-CD49f (BD Biosciences) in PBS containing 5% horse serum overnight at 4°C in a humidified chamber, followed by biotinylated secondary antibodies for 1 hr and fluorescein-avidin D (Vector, Burlingame, CA, USA) or Cy3 anti-avidin (Vector) for 45 min at room temperature. The nuclear staining was accessed using 1 μg/ml DAPI (4',6'-diamidino-2-phenylindole; Sigma) for 1 min. The staining was visualized using a Nikon E-800 microscope equipped with a cooling CCD system (Diagnostic Instruments Inc., Sterling Heights, MI), or under a confocal laser-scanning microscope (Leica TCS SPE).

### Administration of recombinant adenovirus encoding human superoxide dismutase (hSOD), catalase (hCAT) and glutathione peroxidase (hGPx)

Human Cu, Zn-SOD, GPx, or CAT cDNA containing the entire coding sequence was subcloned into the adenovirus shuttle plasmid vector, which contains a promoter of the human phosphoglycerate kinase (PGK) and a polyadenylation signal of bovine growth hormone [22]. Adenoviral administration was followed the procedure as reported previously [23]. Briefly, after the dorsal surface of the spinal cord was compressed by dropping a 10-gm rod from a height of 25 mm (moderate), a 5-μl Exmire microsyringe with a 31-gague needle was positioned at the midline of the cords 2 mm rostral to the contusive center. PBS (no Ad; 2 μl/amimal; n = 3), control Ad (1 × 10^8 ^pfu/μl/animal; n = 3), rAd-SOD (1 × 10^7 ^pfu/animal; n = 3), rAd-CAT (8 × 10^7 ^pfu/animal; n = 3) or rAd-GPx (4 × 10^7 ^pfu/animal; n = 3) was injected 0.8 mm into the dorsal column of the spinal cord within 20 min. Animals were anesthetized with deep pentobarbital, and then perfused with 4% paraformaldehyde in 0.1 M phosphate buffer (pH 7.4). Spinal cords were removed, post-fixed in 4% paraformaldehyde for 3-4 days, and then cryoprotected in 30% w/v sucrose in PBS for 1 day. Approximately 3-4 mm length of the LC (2-3 mm) portion was cut. The tissue block was embedded in OCT medium, and then vertically sectioned at 12 μm thickness. The tissue sections were subjected to in situ apoptotic analysis.

### In situ apoptotic analysis

In situ DNA fragmentation detection kit was purchased from Oncogene (TdT-FragEL TM kit) to study apoptotic cell death. In brief, tissue sections were warmed and dehydrated in PBS. Proteinase K was applied to the tissues followed by 3% H_2_O_2 _in methanol. Terminal deoxynucleotidyl transferase (TdT) was added to the tissues at 37°C for 1.5 hours. The stop solution was then added to terminate the reaction. The apoptotic cells (TdT-FragEL^+ ^cells) were visualized by incubating tissues with DAB, and counted per section.

### Preparation of primary astrocytes and microglia

Media and antibiotics were purchased from Invitrogen (Carlsbad, CA, USA). Cell cultureware and Petri-dishes were obtained from BD Biosciences (San Jose, CA, USA). Fetal bovine serum (FBS) was the product of Hyclone Laboratories (Logan, UT, USA). Primary neuronal and mixed glial cultures were prepared as previously described [19]. In brief, cerebral cortices were removed from embryonic day 17-18 or 1-2-day-old Sprague-Dawley rat brains for neuronal and mixed glial cultures, respectively. The tissue was dissociated in 0.0025% trypsin/EDTA and passed through a 70-μm pore nylon mesh. After centrifugation, the cell pellet was resuspended in DMEM/F-12 (D/F) containing 10% FBS, 50 U/ml penicillin and 50 mg/ml streptomycin. Mixed glial cells (107 cells/flask) were then plated onto poly-D-lysine-coated T75 tissue culture flasks. The medium was renewed every 2-3 days. Eight days later, microglia were collected using shake-off method [20]. The majority of the remaining cells in the culture flask were astrocytes. Astrocytes and microglia were treated with 20 ng/mL of tumor necrosis factor-α and interleukin 1β (T/I; R&D, Minneapolis, MN).

### Injection of chondroitinase ABC (chABC)

The animals received severe SCI, and were treated with chABC (Sigma) right after injury or at day 3 post SCI. The fluid containing 3 μl of PBS (vehicle; n = 4) or chABC (0.03 U/injection, 0.06/rat; n = 4, acute injection; n = 4, delayed injection) were administered by intraspinal injection at the amount of 0.06 U/rat. Briefly, the fluid was injected into approximately 1 mm rostral and caudal to the lesion epicenter. After each injection, the 31-gauge needle was maintained in the spinal cord for an additional 2 min to reduce the possibility of the leakage of the injected fluid from the site. The procedure of animal care was described as above.

### Behavioral Analysis

As previously described [24], animals received either vehicle or chABC were weekly assessed for locomotor function by two blinded observers, using BBB hindlimb locomotor rating scale [20]. Locomotor activities were evaluated by placing animals for 4 min in the open-field with a molded plastic surface. Hindlimb locomotor recovery in animals was scored on the scale of 0 (no hindlimb movement) to 21 (normal mobility).

### Statistical Analysis

The results showing the expression levels of the proteins are presented as mean ± SEM. The two tailed student's *t *test and repeated measures analysis of variance were performed to evaluate the statistical significance of the results (*p *value < 0.05).

## Results

### Comparative protein expression between the acute and chronic injured spinal cord tissues

**Figure 1:**
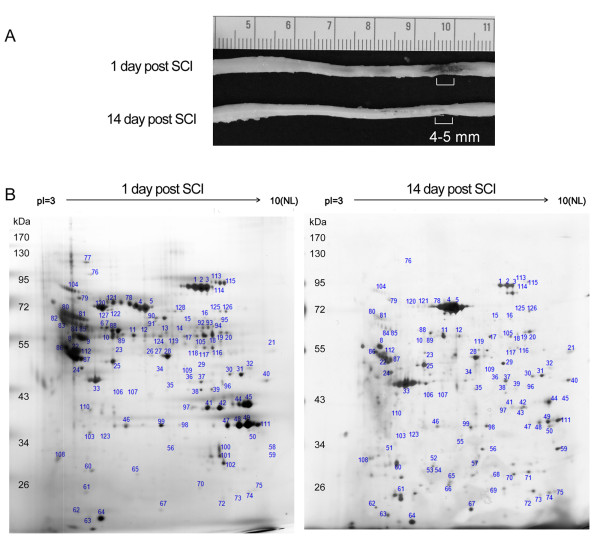
**Proteome analysis of the lesion center of the injured spinal cord**. (A). The injured spinal cords were collected at day 1 (acute) and day 14 (subacute) after SCI. The lesion center (LC) with the length of 4-5 mm was dissected from the injured spinal cord tissues, and subjected to protein extraction for 2-DE. (B). Representative silver stained 2-DE gels show protein spots in the LC of the spinal cord derived from acute and subacute-SCI rats. Protein samples (200 μg) were loaded onto IPG strips (pH 3-10 Non-Linear) and then separated by a 10% SDS-PAGE gel. The gel was stained with silver stain and analyzed. Similar patterns of protein spots on the 2-DE were observed in six independent gels from three different sets of experiments. The spots on the gels were excised, trypsinized, and analyzed by MALDI-TOF-MS as described in Materials and Methods. Protein identification was obtained for 128 protein spots. There were 7 proteins which were biostatistically reduced in the LC at day 14. 12 proteins were found to be significantly upregulated in the LC at day 14 when compared to that detected at day 1 post SCI. Their protein identification and fold change in their expression levels were shown in Table 1 and 2.

The spinal cord tissues were dissected from the LC at day 1 (acute) or day 14 (subacute) post SCI (Figure 1A). Through 2-DE and subjected to MALDI-TOF analysis, we found that protein spots mainly appeared in the section of the pI values 3-10 and the molecular weight was approximately from 20-130 kDa (Figure 1B). An average of 222 protein spots were detected by Image Master 2D analysis software in the acute group and 238 protein spots in the subacute group (Figure 1B). Total 128 proteins were successfully identified through MALDI-TOF mass spectrometry and subsequent database searching (Tables 1, 2, 3 and 4). In comparison to the protein expression in the acute group, quantitative data indicated that the expression intensity of 7 or 12 proteins was biostatistically decreased (Table 1) or increased (Table 2) at least by 1.5-fold in the subacute phase, respectively. However, 42 proteins were considered to have less difference in their expression between day 1 and 14 post SCI (Table 3).

**Table 1:** List of proteins that were down-regulated in the lesion center at day 14 after SCI compared to 1 day after SCI.

Spot **no**.	Function	Protein name	Protein ID	Expression (14d/1d)	14d_mean (SEM)	1d_mean (SEM)	*p *value	Mw/pI	Score	
1~3	acute-phase response	Serotransferrin	TRFE	down	1.241 (0.394)	3.097 (0.627)	0.035	78512/7.14	186	
121	chaperone	Stress-70 protein, mitochondrial	GRP75	down	0.109 (0.032)	0.608 (0.132)	0.008	74097/5.97	64	
102	metabolism	Carbonic anhydrase 1	CAH1	down	ND	0.212 (0.084)	NA	28282/6.86	68	
41, 42, 97	metabolism	Fructose-bisphosphate aldolase C	ALDOC	down	0.440 (0.047)	1.283 (0.263)	0.009	39658/6.67	187	
96	oxidoreduction	Sorbitol dehydrogenase	DHSO	down	ND	0.079 (0.056)	NA	38780/7.14	66	
77	stress response	Heat shock 70 kDa protein 4	HSP74	down	ND	0.193 (0.087)	NA	93997/5.13	127	
78	transport	Hemopexin	HEMO	down	0.360 (0.142)	0.997 (0.123)	0.023	52060/7.58	139	

The proteins changed at least 1.5-fold were listed above. The proteins were downregulated with a *p *value < 0.05. NA, not applicable; ND, non-detectable.

**Table 2:** List of proteins that were up-regulated in the lesion center at day 14 after SCI compared to 1 day after SCI.

Spot **no**.	Function	Protein name	Protein ID	Expression (14d/1d)	14d_mean (SEM)	1d_mean (SEM)	*p *value	Mw/pI	Score
35	actin filament capping	Macrophage-capping protein, Actin regulatory protein CAP-G	CAPG	up	0.239 (0.080)	0.022 (0.021)	0.050	39060/6.11	95
52	actin filament capping	F-actin-capping protein subunit beta	CAPZB	up	0.194 (0.041)	0.025 (0.025)	0.013	30952/5.69	67
110	acute inflammatory response	Haptoglobin	HPT	up	0.132 (0.028)	ND	NA	39052/6.10	63
58,59	cell differentiation	Galectin-3	LEG3	up	0.422 (0.091)	ND	NA	27241/8.59	99
33	cytoskeleton	Beta-actin	ACTB	up	4.016 (0.641)	1.630 (0.260)	0.017	42052/5.29	139
61	GTPase activation	Rho GDP-dissociation inhibitor 1	GDIR1	up	0.486 (0.248)	ND	NA	23450/5.12	106
60	metabolism	Ubiquitin carboxyl-terminal hydrolase isozyme L1	UCHL1	up	1.106 (0.179)	0.393 (0.096)	0.014	25165/5.14	83
22	microtubule	Tubulin beta-5 chain	TBB5	up	0.763 (0.249)	ND	NA	50095/4.78	83
69	oxidoreduction	Flavin reductase	BLVRB	up	0.078 (0.025)	ND	NA	22297/6.49	93
66	oxidoreduction	Heat shock protein beta-1	HSPB1	up	0.201 (0.024)	0.025 (0.002)	0.010	22936/6.12	102
74	oxidoreduction	Superoxide dismutase [Mn], mitochondrial	SODM	up	0.300 (0.045)	0.105 (0.029)	0.020	24887/8.96	64
54	protease inhibitor	Latexin, Endogenous carboxypeptidase inhibitor	LXN	up	0.118 (0.020)	ND	NA	25735/5.77	68

The proteins changed at least 1.5-fold were listed above. The proteins were upregulated with a *p *value < 0.05. NA, not applicable; ND, non-detectable.

**Table 3:** List of proteins that showed a decreased (down) or an increased (up) trend (*p *> 0.05) in the lesion center of the injured spinal cord from the subacute (day 14) SCI group when compared to that detected in the acute (day 1) SCI group.

Spot **no**.	Function	Protein name	Protein ID	Expression (14d/1d)	14d_mean (SEM)	1d_mean (SEM)	*p *value	Mw/pI	Score	
119	actin filament binding	Fascin	FSCN1	down	0.053 (0.022)	0.106 (0.046)	0.406	54474/6.44	184	
6,7	anti-apoptosis	60 kDa heat shock protein, mitochondrial	CH60	down	0.123 (0.050)	0.744 (0.340)	0.052	61088/5.91	96	
11,12	isomerase	Protein disulfide-isomerase A3, p58	PDIA3	down	0.618 (0.188)	1.386 (0.955)	0.344	57044/5.88	80	
13,14	metabolism	D-3-phosphoglycerate dehydrogenase	SERA	down	0.096 (0.081)	0.231 (0.087)	0.355	56457/6.28	90	
38	metabolism	Acetyl-CoA acetyltransferase, cytosolic	THIC	down	0.052 (0.009)	0.189 (0.112)	0.272	41538/6.86	60	
44,45	metabolism	Fructose-bisphosphate aldolase A	ALDOA	down	1.256 (0.239)	1.905 (0.570)	0.292	39783/8.31	125	
46	metabolism	L-lactate dehydrogenase B chain	LDHB	down	0.396 (0.072)	1.092 (0.613)	0.283	36874/5.70	71	
99	metabolism	Malate dehydrogenase, cytoplasmic	MDHC	down	0.240 (0.087)	0.648 (0.238)	0.121	36117/8.93	217	
100, 101	metabolism	Carbonic anhydrase 2	CAH2	down	0.053 (0.025)	0.484 (0.228)	0.071	29096/6.89	76	
109	metabolism	Glycine amidinotransferase, mitochondrial	GATM	down	0.032 (0.011)	0.052 (0.016)	0.416	48724/7.17	146	
113~ 115	metabolism	Aconitate hydratase, mitochondrial	ACON	down	0.164 (0.058)	0.712 (0.323)	0.102	86121/7.87	183	
39	metabolism	Creatine kinase M-type	KCRM	down	0.066 (0.020)	0.101 (0.032)	0.391	43246/6.58	63	
30,31	metabolism	Phosphoglycerate kinase 1	PGK1	down	0.452 (0.123)	0.803 (0.468)	0.446	44909/8.02	116	
9,10	microtubule	Tubulin alpha-1B chain	TBA1B	down	1.111 (0.488)	1.941 (0.876)	0.370	50120/4.94	86	
89	microtubule	Tubulin alpha-1A chain	TBA1A	down	0.146 (0.039)	0.279 (0.062)	0.102	50788/4.94	135	
88,90, 91	neurogenesis	Dihydropyrimidinase-related protein 2	DPYL2	down	0.176 (0.088)	0.313 (0.116)	0.366	62638/5.95	105	
92~94	neuron differentiation	Dihydropyrimidinase-related protein 5	DPYL5	down	0.061 (0.046)	0.319 (0.189)	0.132	61501/6.60	117	
105	oxidoreduction	Dihydrolipoyl dehydrogenase, mitochondrial	DLDH	down	0.151 (0.069)	0.376 (0.165)	0.214	54574/7.96	84	
95	oxidoreduction	Catalase	CATA	down	0.028 (0.027)	0.056 (0.013)	0.364	59719/7.07	187	
82,83	protease inhibitor	Serine protease inhibitor A3K	SPA3K	down	0.299 (0.078)	0.866 (0.289)	0.073	46532/5.31	113	
15	protein assembly	Stress-induced-phosphoprotein 1, Hsc70/Hsp90-organizing protein	STIP1	down	0.035 (0.020)	0.172 (0.139)	0.288	63158/6.40	65	
17	proteolysis	Cytosol aminopeptidase	AMPL	down	0.122 (0.040)	0.188 (0.064)	0.400	56514/6.77	62	
120	stress response	Heat shock cognate 71 kDa protein	HSP7C	down	0.391 (0.257)	0.865 (0.332)	0.288	71055/5.37	123	
119	actin filament binding	Fascin	FSCN1	down	0.053 (0.022)	0.106 (0.046)	0.406	54474/6.44	184	
63	anti-apoptosis	Lactoylglutathione lyase	LGUL	up	0.488 (0.078)	0.231 (0.090)	0.074	20977/5.12	67	
62	cell proliferation	Translationally-controlled tumor protein	TCTP	up	0.181 (0.051)	0.058 (0.057)	0.216	19564/4.76	128	
8	chaperone	Protein disulfide-isomerase	PDIA1	up	0.897 (0.253)	0.263 (0.099)	0.097	57315/4.82	197	
67	chaperone	Protein DJ-1	PARK7	up	0.260 (0.087)	0.113 (0.034)	0.198	19961/6.32	68	
124	chaperone	T-complex protein 1 subunit beta	TCPB	up	0.078 (0.015)	0.031 (0.030)	0.190	57422/6.01	76	
21	metabolism	Elongation factor 1-alpha 1	EF1A1	up	0.631 (0.273)	0.160 (0.055)	0.142	50424/9.10	77	
29	metabolism	Isocitrate dehydrogenase [NADP]	IDHC	up	0.113 (0.111)	0.056 (0.044)	0.679	47047/6.53	108	
50	metabolism	L-lactate dehydrogenase A chain	LDHA	up	0.209 (0.045)	0.080 (0.028)	0.058	36712/8.45	64	
53	metabolism	Dimethylarginine dimethylaminohydrolase 2	DDAH2	up	0.118 (0.044)	0.015 (0.014)	0.158	30011/5.66	116	
73,75	oxidoreduction	Peroxiredoxin-1	PRDX1	up	0.684 (0.378)	0.076 (0.036)	0.158	22323/8.27	91	
65	oxidoreduction	Peroxiredoxin-6	PRDX6	up	0.199 (0.104)	0.074 (0.023)	0.359	24860/5.64	74	
79	protein assembly	78 kDa glucose-regulated protein	GRP78	up	0.323 (0.116)	0.136 (0.045)	0.245	72473/5.07	285	
51	proteolysis	Cathepsin B	CATB	up	0.245 (0.081)	0.159 (0,158)	0.619	38358/5.36	72	
106, 107	proteolysis	Cathepsin D	CATD	up	0.840 (0.373)	0.036 (0.026)	0.075	45165/6.66	132	
64	signal transduction	Phosphatidylethanolamine-binding protein 1	PEBP1	up	1.539 (0.440)	0.910 (0.214)	0.277	20902/5.48	82	
72	signal transduction	GTP-binding nuclear protein Ran	RAN	up	0.184 (0.090)	0.116 (0.055)	0.606	46532/5.31	113	
57	stress response	Endoplasmic reticulum protein ERp29	ERP29	up	0.118 (0.091)	0.029 (0.029)	0.513	28614/6.23	63	
56	ubiquitin-dependent protein catabolism	Proteasome subunit alpha type-1	PSA1	up	0.177 (0.038)	0.091 (0.027)	0.162	29784/6.15	64	

### Expression of oxidoreduction-related proteins in the injured spinal cord in the subacute phase

**Figure 2:**
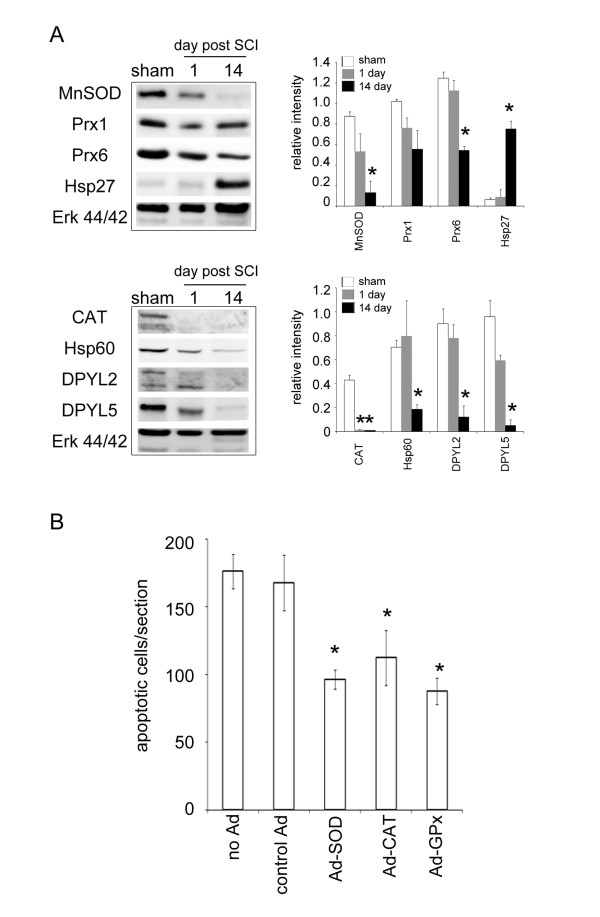
**Expression of stress proteins and antioxidant enzymes in the lesion center**. (A) Western blot analysis shows time course change in the expression levels of MnSOD, Prx1, Prx6, Hsp27, catalase (CAT), Hsp60, DPYL2 and DPYL5. The proteins extracted from the LC of the injured spinal cords at the different survival time points (day 1 and 14) after SCI or sham control. The same blot was stripped and reprobed with anti-ERK44/42 antibody as internal loading control. Relative intensity of the indicated protein level bands normalized to ERK 44 was measured. Data are presented as means ± SEM from three separate experiments. *p < 0.05 versus sham control. (B). The spinal cord was removed at day 8 post SCI from the rats without or receiving control Ad, rAd-SOD, rAd-calatase (CAT), rAd-GPx gene therapy. Horizontal spin cord tissue sections were subjected to in situ apoptosis analysis. Data represent the mean ± SEM. *p < 0.05 versus the control group without Ad injection. ^#^*p *< 0.05 versus the group treated with control Ad.

An increase in Hsp27 (HSPB1; spot 66) at day 14 after SCI was observed by proteomic analysis (Table 2) and western blotting (Figure 2A). As shown in Table 3, the proteomic analysis indicated that the expression of DPYL2 (spot 88, 90 and 91), DPYL5 (spot 92-94), and heat shock protein 60 (CH60/Hsp60; spot 6 and 7) in the LC at day 14 post SCI was reduced when compared to that detected at day 1. Although no significant difference in the intensity of peroxiredoxin 1(Prx1; spot 73 and 75) and Prx6 (spot 65) was seen in the LC between day 1 and day 14 (Table 3), western blot analysis showed that these proteins were time-dependently reduced post SCI (Figure 2A).

The protein spot 74 in 2DE gel was identified as MnSOD. After normalization by the total volume of the protein spots indicated in 2DE gel, its relative intensity levels in the LC collected at day 14 post SCI was much higher than that measured at day 1 (Table 2). Western blot analysis was performed to ensure the levels of MnSOD in the LC at the two survival time points. Unexpectedly, the levels of MnSOD were found to reduce at day 14 when compared to that seen in the sham control or injured tissue at day 1 post SCI (Figure 2A). The findings from western blotting showing a decreased level of MnSOD in the LC at day 14 were confirmed by immunofluorescence (see Additional File 1; Figure S1). We also noticed that the levels of Cu, Zn-SOD was reduced in the LC at day 14 post SCI, whereas GPx was expressed in the sham-operated and injured spinal cord (see Additional File 1; Figure S1). In comparison with that observed in the LC at day 1 post SCI, catalase (CAT; spot 95) had a decreased trend at day 14 (Table 3). The observation from the proteomic analysis was confirmed by western blot analysis (Figure 2A).

Given the fact that the reduction of the antioxidant enzymes in the LC at day 1 and day 14 post SCI compared to that in sham-operated tissues (Figure 2A), we examined whether the neural cell survival was increased after gene transfer of antioxidant enzymes (SOD, CAT, and GPx) via adenoviral vector right after SCI. In parallel, we conducted rAd-GFP gene transfer into the contused spinal cord to evaluate the efficacy of intraspinal injection of recombinant adenovirus. Most neural cells in the injured spinal cord were transduced by rAd-GFP (see Additional File 2; Figure S2). In situ apoptotic analysis showed that rAd-SOD, rAd-CAT, and rAd-GPx, but not control Ad, significantly reduced the number of apoptotic cells in the injured spinal cord compared to those found in the injured spinal cord without any treatment (Figure 2B).

### Extensive inflammation in the injured spinal cord in the subacute phase

**Figure 3:**
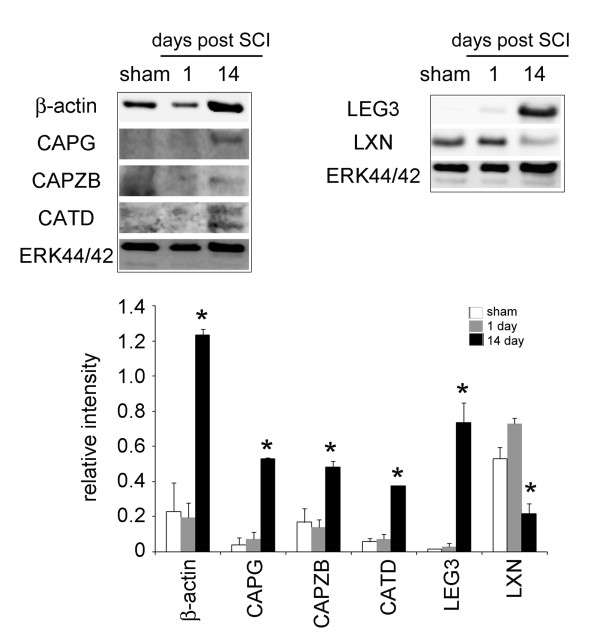
**Expression of structure proteins and inflammatory regulator proteins in the lesion center**. Western blot analysis showed time course change in the expression levels of β-actin, CAPG, CAPZB, CATD, galectin-3 (LEG3) and LXN. The proteins extracted from the LC of the injured spinal cords at the different survival time points (day 1 and 14) after SCI or from sham control. The same blot was stripped and reprobed with ant-ERK44/42 antibody as internal loading control. Relative intensity of the indicated protein level bands normalized to ERK 44 was measured. Data are presented as means ± SEM from three separate experiments. *p < 0.05 versus sham control.

**Figure 4:**
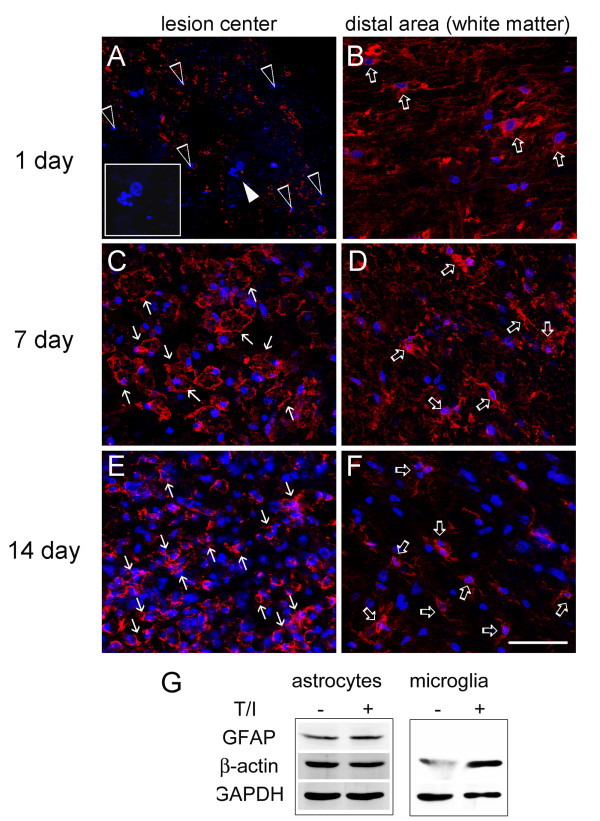
**Immunofluorescence for β-actin expression in injured spinal cord tissue sections**. The injured spinal cord tissue sections were collected at day 1 (A,B), day 7(C,D), and day 14(E,F) after SCI, and then subjected to immunofluorescence for β-actin (red). The tissues were also subjected for nuclear staining using DAPI (blue). In general, cell debris with β-actin immunoreactivity (open arrowheads) and fragmented/damaged nuclei (inset in A) were exclusively detected in the lesion center (LC) at 1 day post SCI. However, numerous β-actin^+ ^cells (arrows) were accumulated in the LC at day 7 and 14 post SCI. Noted that β-actin^+ ^cells (open arrows) were detected in the distal area (white matter) to the LC at day 1, 7 and 14 post SCI. Scale bar in A-F, 50 μm. (G) Primary astrocytes and microglia prepared from neonatal rat cortical tissues were treated for 24 hours with proinflammatory factor, TNF-α and IL-1β (T/I) at the doses of 20 ng/ml. The total proteins were extracted, separated by SDS-PAGE, and analyzed by western blotting with anti-β-actin, anti-GFAP.

**Figure 5:**
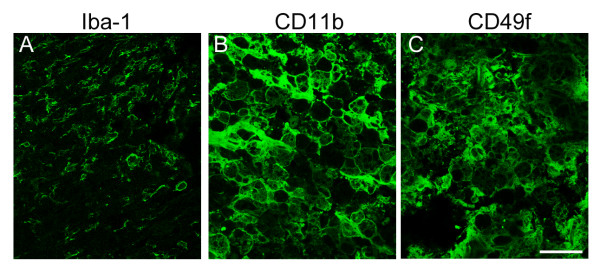
**Immunofluorescence examination of Iba-1**^**+ **^**microglia, CD11b**^**+ **^**macrophages, CD49f**^**+ **^**macrophages/monocytes in the lesion center at day 14 post DSCI**. The injured spinal cord tissue sections were collected at day 14 after SCI, and then subjected to immunofluorescence for Iba-1 (A), CD11b (B), or CD49f (C). Noted that the Iba1^+ ^cells shown in A were located in the peritraumatic zone, and CD11b^+^/CD49^+ ^cells in B and C were situated in the lesion center. Scale bar in A-F, 50 μm.

We noticed that β-actin (spot 33) and β-tubulin 5 (spot 22) was biostatistically increased in the LC at day 14, when compared to that detected at day 1 (Figure 1B and Table 2). The intensity of actin filament capping proteins, CAPG (spot 35) and CAPZB (spot 52), were also found increased in 2-DE (Table 2). Western blot analysis also verified that β-actin, CAPG and CAPZB were dramatically increased in the LC at day 14 post SCI (Figure 3). Immunofluorescence also confirmed that β-actin^+ ^cells with an irregular morphology accumulated exclusively in the LC at day 7 and 14 post SCI (Figure 4C, E), while β-actin^+ ^cell debris was detected in the LC at day 1 post SCI (Figure 4A). DAPI nuclei staining indicated that extensive cell death was observed at day 1 post SCI (Figure 4A). We also noticed that β-actin^+ ^cells with a hypertrophic morphology were found at day 1 post SCI in the white matter of the spinal cord distal to the LC(Figure 4B), whereas ramified β-actin^+ ^cells were observed at day 7 and day 14 post SCI (Figure 4D, F). Through proteomic approach, we found that the regulators of inflammation and carboxypeptidase inhibitor, galectin-3 (LEG3; spot 58,59) and latexin (LXN; spot 54), were increased in the LC at day 14 post SCI (Table 2). By Western blot analysis, an increase in LEG3, but not LXN, was found along the longer survival time points (Figure 3). Cathepsin D (CATD), one of lysosomal enzymes enriched in macrophages, was also increased in the LC at day 14 post SCI (Table 2 and Figure 3). Furthermore, immuofluorescence indicated that Iba-1^+ ^microglia were accumulated in the proximal site to the LC (Figure 5A). Moreover, CD11b (Mac-1) or CD49f-positive macrophages were observed in the LC (Figure 5B, C) and the proximal area to the LC at day 14 post SCI (data not shown).

Alternatively, in vitro study using primary rat glial cultures also showed that the proinflammatory cytokines, TNF-α and IL-1β (T/I), did increase the expression of β-actin protein levels in primary microglia (Figure 4G). However, only a slight change was detected in the expression of β-actin protein levels in primary microglia with or without T/I treatment. Thus, the β-actin^+ ^cells detected in the LC could be mainly inflammatory cells either derived from resident microglia or infiltrating monocytes/leukocytes from the periphery blood, and they could produce proinflammatory cytokines to increase the β-actin protein levels in glial cells (such as astrocytes) in the injury penumbra.

### Delayed treatment with chondroitinase ABC in hindlimb locomotion recovery after SCI

**Figure 6:**
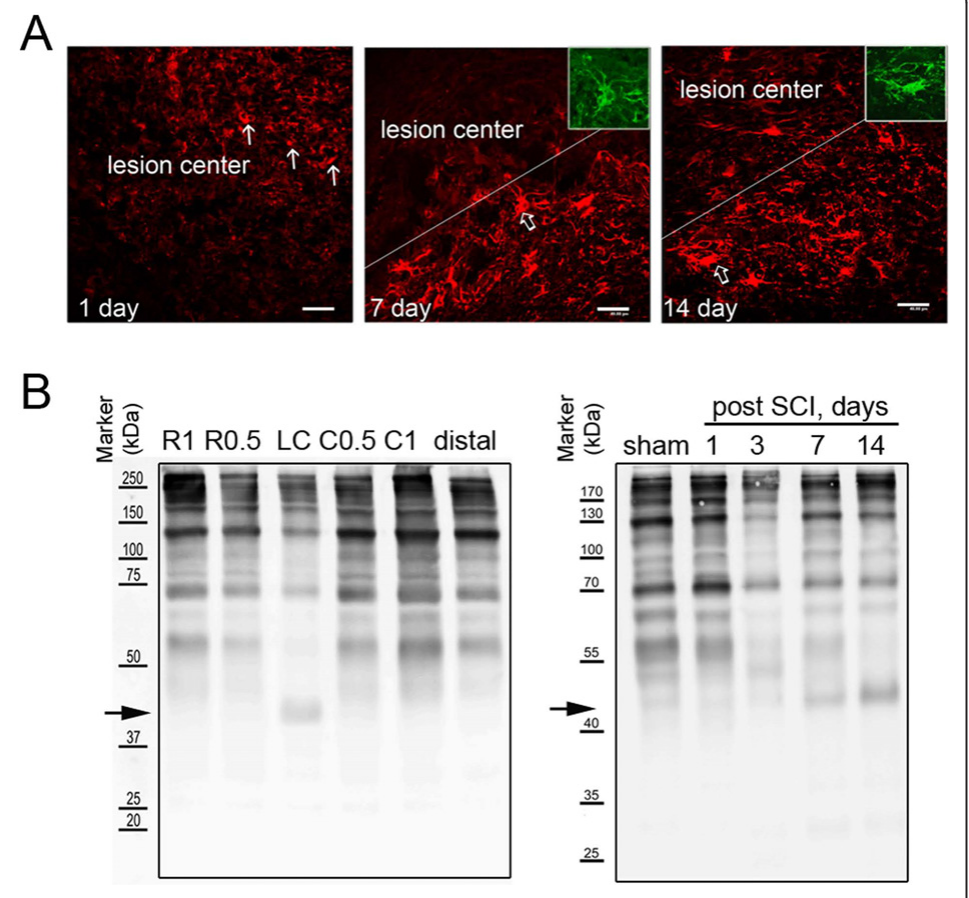
**Time expression prolife of GFAP and CSPGs in the injured spinal cord**. (A) The injured spinal cord tissue sections were collected at day 1, 7, and 14 after SCI. The tissue sections were subjected to immunofluorescence for GFAP (red). GFAP^+ ^stellated cells (open arrows) were observed in the areas proximal to the lesion center (LC) or migrated into the LC at day 7 and 14 after SCI, and the GFAP^+ ^cells were also immunoreactive with β-actin (insets). Note that cell debris with GFAP immunoreactivity (arrows) was detected in the LC at 1 day post SCI. Scale bar, 40 μm. (B). Total proteins were prepared from injured spinal cord tissues 0.5 or 1 mm rostral (r0.5 and r1) and caudal (c0.5 and c1) to the injury epicenter (LC) at day 31 post SCI (left panel). Sham-operated control was from animal only receiving laminectomy. Alternatively, the proteins were extracted from the lesion centers of spinal cord tissues at day 1, 3, 7 and 14 post SCI (right panel). The sample preparation was described in Materials and Methods. To examine the levels of CSPGs in the injured spinal cord tissues, western blotting was then performed using anti-chondroitin-4-sulfate antibody. Arrows indicate CSPGs or CSPG fragments with 40 kDa approximately.

**Figure 7:**
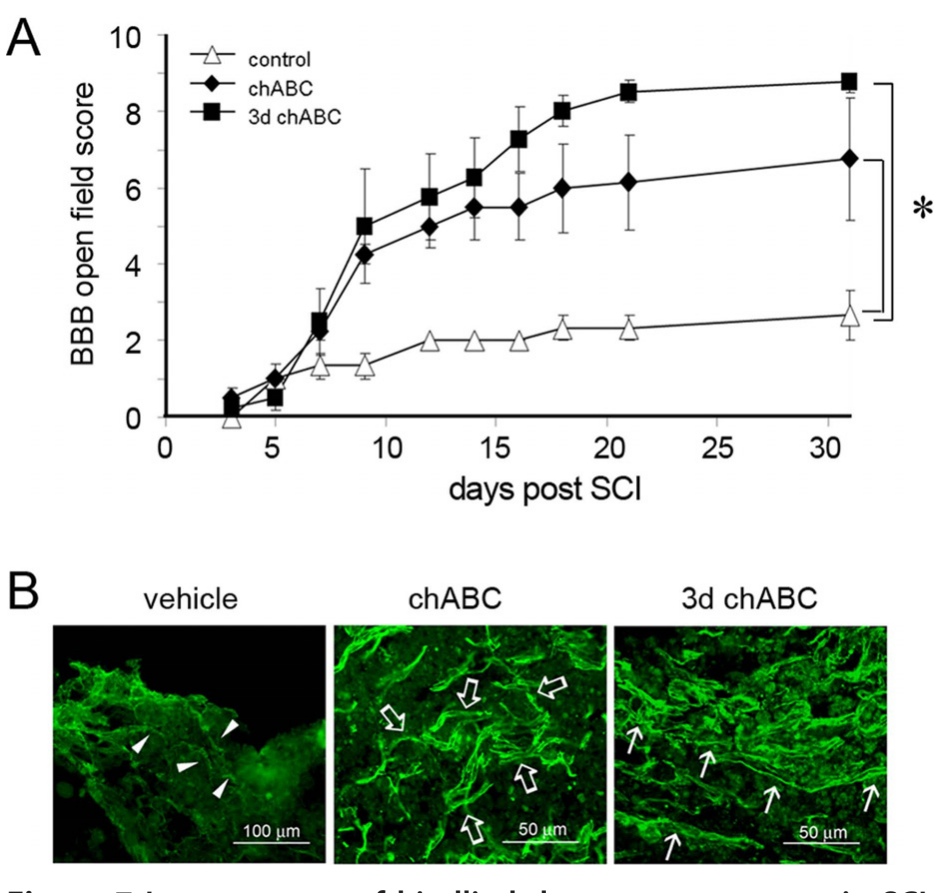
**Improvement of hindlimb locomotor recovery in SCI rats by treatment with chABC**. (A). Rats received administration with vehicle (n = 4), or with chABC (0.03 U/injection, 0.06 U/rat) immediately after SCI (n = 4) or at 3 day post SCI (n = 4). To examine hindlimb locomotor function in SCI rats, the BBB open-field analysis was conducted. Data are presented as mean ± SEM. *p <0.05 versus the vehicle-treated group. (B). The spinal cord tissues were collected from SCI rats at day 31. The longitudinal tissue sections containing the LC were subjected to immunofluorescence for GAP-43. Arrowheads indicate the fine fragmented GAP-43-positive neuronal fibers in the vehicle-treated control. Arrows and open arrows show the GAP-43-positive neuronal bundles in chABC-treated group. Scale bar, 100 μm (left panel) and 50 μm (middle and right panels).

We noticed no change in the levels of astrocytic proteins, GS (spot 36 and 37) and GFAP (spot 87) in the LC at day 1 and 14 post SCI (Table 4). Immunofluorescence indicated that GFAP^+ ^cell fragments were observed at the lesion site at day 1 post SCI (Figure 6A), while GFAP^+ ^hypertrophic astrocytes were detected in the injury penumbra at day 7 and 14 post SCI. These GFAP^+ ^cells were also colocalized to β-actin^+ ^cells (Figure 6A, insets). In addition, we observed that few GFAP^+ ^astrocytic processes invaded to the LC (Figure 6A) at day 14 post SCI. The results from immunofluorescence explain that comparable GFAP detected by proteome analysis in the LC at day 14 was derived from invading astrocytes, which is the pathophysiological event proposed in SCI [1]. Given the fact that glial scar is mainly formed by chondroitin sulfate proteoglycans (CSPGs) primarily produced by reactive astrocytes, the production of CSPGs at the different spinal cord tissue blocks was examined at day 31 after SCI. As shown in Figure 6B, there were differential levels of CSPGs detected in the spinal cord tissues rostral and caudal to the lesion center. However, CSPGs approximately corresponding to 40- kDa were only present in the LC. In addition, the 40-kDa CSPGs were initially detected in the LC at day 3, continued to be seen at day 7 and 14 post SCI (Figure 6B). Based on the spatial and temporal levels of 40-kDa CSPGs in the injured spinal cord, injection into the injured spinal cord with chABC at the different time points post SCI was performed. The hindlimb locomotor function was assessed every 2-3 days up to 31 days using BBB locomotor rating scale. Through the evaluation of behavior analysis, we found that administration of chABC right after SCI or at day 3 post SCI enhanced the hindlimb locomotion in rats with SCI (Figure 7A). However, at day 31 after SCI, BBB scores in rats receiving delayed treatment with chABC were higher than that observed in animals with acute treatment with chABC. Immunofluorescence showed that there were numerous neuronal fiber bundles with GAP-43-positive staining in the injured spinal cord receiving chABC immediately after SCI or by delayed treatment with chABC (Figure 7B, arrows), whereas only numerous fine fragmented neuronal fibers remained in the injured spinal cord without treatment (Figure 7B, arrowheads). In addition, when compared to GAP-43 immunostaining on the LC with acute chABC treatment, there was more elongated GAP-43-positivie neuronal fiber bundles present in the injured center of the spinal cord with delayed treatment by chABC (Figure 7B).

**Table 4:** List of proteins that were changed less than 1.5-fold in the lesion center of the injured spinal cord from the subacute (day 14) SCI group when compared to that detected in the acute (day 1) SCI group.

**no**.	function	protein name	protein ID	14d_mean (SEM)	1d_mean (SEM)	*p *value	Mw/pI	score
128	actin binding	WD repeat-containing protein 1	WDR1	0.036 (0.035)	0.034 (0.017)	0.980	66824/6.15	76
80	acute phase	T-kininogen 2	KNT2	0.648 (0.352)	0.950 (0.085)	0.481	48757/5.94	102
81	acute phase	T-kininogen 1	KNT1	0.270 (0.040)	0.202 (0.055)	0.353	48828/6.08	144
87	cytoskeleton	Glial fibrillary acidic protein	GFAP	0.458 (0.093)	0.535 (0.036)	0.469	49927/5.35	144
16	metabolism	Bifunctional purine biosynthesis protein PURH	PUR9	0.039 (0.009)	0.056 (0.055)	0.629	64681/6.69	70
18~20	metabolism	Pyruvate kinase isozymes M1/M2	KPYM	0.719 (0.280)	0.864 (0.433)	0.778	58294/6.63	134
24	metabolism	Gamma-enolase	ENOG	0.343 (0.130)	0.446 (0.114)	0.581	47111/5.03	155
25	metabolism	Creatine kinase B-type	KCRB	0.996 (0.226)	0.767 (0.177)	0.469	42983/5.30	151
26~28	metabolism	Alpha-enolase	ENOA	0.714 (0.271)	1.040 (0.364)	0.486	47440/6.16	185
32	metabolism	3-ketoacyl-CoA thiolase, mitochondrial	THIM	0.133 (0.040)	0.153 (0.061)	0.782	42244/8.09	137
36,37	metabolism	Glutamine synthetase	GLNA	0.338 (0.096)	0.249 (0.080)	0.505	42982/6.64	179
47~49	metabolism	Glyceraldehyde-3-phosphate dehydrogenase GAPDH	G3P	2.351 (0.603)	3.215 (1.492)	0.577	36090/8.14	88
68,70, 71	metabolism	Triosephosphate isomerase	TPIS	0.609 (0.219)	0.413 (0.134)	0.461	27345/6.89	164
36,37	metabolism	Glutamine synthetase	GLNA	0.338 (0.096)	0.249 (0.080)	0.505	42982/6.64	179
111	metabolism	Malate dehydrogenase, mitochondrial	MDHM	0.874 (0.241)	0.726 (0.240)	0.682	36117/8.93	217
123	metabolism	Pyruvate dehydrogenase E1 component subunit beta, mitochondrial	ODPB	0.103 (0.059)	0.070 (0.002)	0.636	38957/6.20	100
125, 126	metabolism	Transketolase	TKT	0.143 (0.047)	0.147 (0.081)	0.970	67601/7.23	92
103	microtubule	Tubulin alpha-1C chain	TBA1C	0.132 (0.049)	0.098 (0.021)	0.559	49905/4.96	64
112	microtubule	Tubulin beta-2C chain	TBB2C	1.464 (0.416)	1.289 (0.343)	0.764	50225/4.79	210
43	oxidoreduction	Alcohol dehydrogenase [NADP+]	AK1A1	0.153 (0.030)	0.135 (0.105)	0.860	36711/6.84	84
98	oxidoreduction	Aldose reductase	ALDR	0.128 (0.047)	0.124 (0.042)	0.956	35774/6.26	101
116~ 118	oxidoreduction	Glutamate dehydrogenase 1, mitochondrial	DHE3	0.286 (0.100)	0.410 (0.233)	0.613	61719/8.05	172
86	protease inhibitor	Serine protease inhibitor A3N	SPA3N	0.751 (0.144)	0.748 (0.369)	0.995	46622/5.33	109
23	proteolysis	Cytosolic non-specific dipeptidase	CNDP2	0.174 (0.029)	0.176 (0.083)	0.983	53116/5.43	120
34	proteolysis	Aminoacylase-1A	ACY1A	0.058 (0.014)	0.074 (0.031)	0.628	46060/6.03	77
104	secreted glycoprotein	Alpha-1B-glycoprotein	A1BG	0.197 (0.070)	0.211 (0.104)	0.914	57127/6.89	76
84,85	signal transduction	Rab GDP dissociation inhibitor alpha	GDIA	0.403 (0.118)	0.440 (0.112)	0.829	50504/5.00	73
122	stress response	Heat shock-related 70 kDa protein 2	HSP72	0.084 (0.031)	0.100 (0.024)	0.738	69599/5.51	115
4,5	transport	Serum albumin	ALBU	3.629 (1.939)	4.722 (1.882)	0.722	70682/6.09	306
40	transport	Aspartate aminotransferase	AATM	0.246 (0.070)	0.165 (0.041)	0.387	47683/9.13	64
55	transport	3-mercaptopyruvate sulfurtransferase	THTM	0.053 (0.014)	0.047 (0.046)	0.893	33205/5.88	75
108	transport	Clathrin light chain B	CLCB	0.310 (0.090)	0.324 (0.156)	0.953	25216/4.65	63
127	transport	V-type proton ATPase catalytic subunit A	VATA	0.056 (0.028)	0.057 (0.027)	0.982	68283/5.42	77

## Discussion

Previous studies using genomics or proteomics approach have focused on changes in gene expression and protein production in the injured spinal cord within a week after SCI [2,15,16,21]. To comprehend pathophysiological changes at the longer survival time points after SCI, we examined protein expression changes in the LC at day 14 post severe SCI on spinal T9/10 segment. The genomic study indicates that inflammation-related transcription factors and cytokines were upregulated at the acute phase after SCI [2]. In the proteome-based study, Prx2, HspA1B, cytoskeleton reorganization-related proteins (such as neurofilament light chain, annexin 5, tubulin beta, peripherin, GFAP, septin 7) were increased during the first week after SCI when compared to sham-operated control [15,16]. Here, we demonstrate that oxidoreduction-related enzymes (Prx1, Prx6, MnSOD and CAT) and Hsp60 were reduced at day 14 post SCI, while β-actin and actin-capping proteins (CAPG and CAPBZ) were increased. Similar to their expression levels found in the contused spinal cord within one week post SCI [16], Hsp27 and LEG3 were also increased in the LC at day 14.

An increase in the production of Prx1, Prx6 and MnSOD in the LC at day 14 was found by the proteomic analysis when compared to that at day 1 post SCI. However, through western blot analysis, the levels of the three proteins were significantly reduced in the LC at day 14. The apparent differences between proteome data and western blot findings could be attributed to the following issues in the proteomic analysis. First, the protein samples isolated from tissues at day 1 and day 14 were electrophoresized in the separate gels. Thus, the variation could occur during sliver staining process, although the intensity of a protein spot in 2D gel was normalized by the sum of the intensity of all the spots present in each gel. Second, it is known that there is the difficulty to obtain similar resolution in the alkaline ranges of IPGs [25,26]. As shown in Figure 1B, the poor focusing for the three protein spots in 2DE could gain inaccurate quantification of their spot intensity. Third, the three proteins could be modified in the LC under extensive oxidative stress at day 1 post SCI, since Prx1/6 and MnSOD have been known to be modified by phosporylation, oxidation, or nitration, respectively [27-31]. The protein modification would change their *p*I values and affect their mobility in the IPGs. Although the phosphorylated/oxidized form of Prx1/6 and the nitrated form of MnSOD in 2DE remain to be identified, the intensities of the three protein spots shown in 2DE would not be sufficient to indicate the exact production of the three proteins.

The upregulation of Hsp27 gene expression and protein production at 24 h post SCI have been reported [21,32]. Through proteomic and western blot assays, we also found that Hsp27 was exclusively increased at day 14 post severe SCI (Table 2; Figure 2), indicating the involvement of Hsp27 in prolonged inflammation occurring in the late phase of SCI. Nevertheless, the exact role of Hsp27 in SCI-induced neuropathogenesis remains to be explored. On the other hand, western blotting indicated that the decline in the levels of MnSOD, CAT, Prx1 and Prx6 was observed at day 14 when compared to than those observed in the sham control and injured tissues collected at 1 day post SCI. The observations demonstrate that the production of oxidoreduction-related enzymes was downregulated in the LC in the subacute phase post SCI. The reduction in these oxidoreduction-related proteins might be due to the extensive cell death occurring in the subacute phase. However, we could not rule out the possibility that the decline in the expression of oxidoreduction-related proteins described as above might lead to neural cell death exclusively occurred in the LC in the subacute phase.

LEG3, the regulator of inflammation, has been found to be induced in the contused spinal cord at 3 and 7 day after SCI, and in cultured microglia upon stimulation [33]. The molecule is considered to be involved in the phagocytosis of degenerated myelin or other tissue debris in the injured CNS [34]. From our proteomic profile and western blot analysis, the expression of LEG3 was exclusively increased in the injured spinal cord at day 14 post SCI, implying that active phagocytosis takes place in the LC over two weeks post SCI. Under harmful conditions (such as oxidative stress and inflammation), the ubiquitin/proteasome pathway is required for removal of abnormal proteins in the cells to maintain neuronal homeostasis [35]. In addition, the upregulation of lysosomal cysteine proteases (such as cathepsins) are associated with various neurological disorders [36]. For instance, an increase in CATD has been shown in several neuropathological disorders, including amyotrophic lateral sclerosis [37], and autoimmune encephalomyelitis [38]. Recent study has also shown that CATD was found increased in the subacute phase after clip spinal cord compression injury [39]. The immunohistochemical observations suggest that CATD could play an important role in the phagocytosis and lysosomal activation in macrophages/microglia during trauma-induced neuroinflammation [39]. In consistence with the findings described as above, we have shown that CATD was about 23-fold increased in the LC at day 14 SCI when compared to that detected at day 1 after SCI (Table 1). In addition, this increase in the levels of CATD expression at day 14 post SCI was verified by western blot analysis (Figure 3). In conjunction with findings for oxidoreduction related proteins (Hsp60, Prx1/6, MnSOD, and CAT), galectin-3, antioxidant enzyme gene transfer, and β-actin immunofluorescence, the data underscore the fact that intense oxidative stress, inflammation and glial activation sustain at the late phase of SCI.

The microenvironment constituents (such as extracellular matrix molecules) after SCI are seriously altered along with massively increased expression of proteins related to inflammation, stress and cell death. For instance, CSPGs are highly accumulated in the injured spinal cord surrounding the LC [40,41]. We observed that multiple CSPGs are present in the injured spinal cord rostral (R0.5 and R1) and caudal (C0.5 and C1) to the LC at day 31, although the CSPG molecules with approximately 40 kDa was observed only in the LC at day 3, 7, 14 and 31 post SCI. Due to the fact that CSPGs are mainly secreted by glial cells, profound astrogliosis could initiate at day 7 post SCI. Infusion of chABC has been found to effectively prune CSPGs in the injured spinal cord, which improves axonal extension toward the brain [42,43]. Owing to the fact that gliosis is needed to prevent inflammation progress toward the remaining tissues [1], acute treatment with chABC possibly inducing detrimental effect on tissue repair is considered. Although a bolus of acute or delayed injection with chABC improved hindlimb locomotion in rats with severe SCI, less variation in mean of BBB scores was observed in the animal group receiving delayed chABC when compared to that seen in the group with acute chABC treatment. Acute treatment with chABC has been reported to induce better axonal outgrowth in the injured CNS [44]. It has also been reported that animals receiving acutely treatment with chABC improved skilled forelimb reaching [45]. A previous study has shown efficient improvement on locomotion recovery in rats by delayed ventricular injections of chABC after stabbing injury at spinal C4 segment, although the group received acute injection by chABC had better outcomes [45]. Similarly, our findings provided further information that a bolus of delayed intraspinal injection with chABC effectively enhances hindlimb locomotion recovery in rats with SCI.

## Conclusions

In summary, our study demonstrates that the proteomic changes in the LC at the subacute SCI phase are consistent with the SCI-induced pathological outcome. These changes reflect the fact that the progressive inflammation within two weeks after SCI may promote gliosis. The data provide a resource for the proteomic profiles of the contused spinal cord tissue at the subacute SCI phase, which supports the therapeutic interventions based on a sustainable supply of anti-oxidative molecules to inhibit the progress of inflammation and oxidative stress after SCI.

## Competing interests

The authors declare that they have no competing interests.

## Authors' contributions

CYW and SFT conceived the study, designed experiments, and drafted the manuscript. JKC, YTW, and CSY conducted Protein identification by mass spectrometer by mass spectrometer analysis. MJT and SKS carried out the preparation of recombinant adenovirus. All authors read and approved the final manuscript.

## Supplementary Material

Additional file 1 **Figure S1: Examination of MnSOD, Cu,ZnSOD and GPx expression in the lesion center at day 1 and day 14 post SCI**. (A) The injured spinal cord tissue sections were collected at day 1 and day 14 (E,F) after SCI, and then subjected to immunofluorescence for MnSOD. There were numerous MnSOD^+ ^cells observed in the lesion center at day 1 post SCI, while few MnSOD^+ ^cells were found in the lesion center at day 14. Scale bar, 50 μm. (B) Western blot analysis showed the reduction of Cu,ZnSOD and GPx levels at the lesion center when compared to that detected in the sham control. The proteins extracted from the lesion center of the injured spinal cords at the different survival time points (day 1, 7 and 14) after SCI or from sham control. The same blot was stripped and reprobed with ant-ERK44/42 antibody as internal loading control. Click here for file

Additional file 2 **Figure S2: Green fluorescent protein (GFP) expression in ependymal cells lining along the central canal of the spinal cord and in neural cells located at the dorsal and ventral portions of the injured spinal cord**. rAd-GFP (1 × 10^8 ^pfu/injection) was injected into the rat spinal cord 1 mm rostral to the lesion center within 10 minutes after SCI. The rats were sacrificed at 7 days post SCI, and perfused in 4% paraformaldehyde. The injured spinal cord tissues were removed and prepared for cryostat as described in Experimental Section. Scale bar, 25 μm. Click here for file
