# Supplementary figures and images for: Reduction in antioxidant enzyme expression and sustained inflammation enhance tissue damage in the subacute phase of spinal cord contusive injury
Source: J Biomed Sci. 2011 Feb 7;18(1):13. doi: 10.1186/1423-0127-18-13 (PMC3040708; doi:10.1186/1423-0127-18-13)

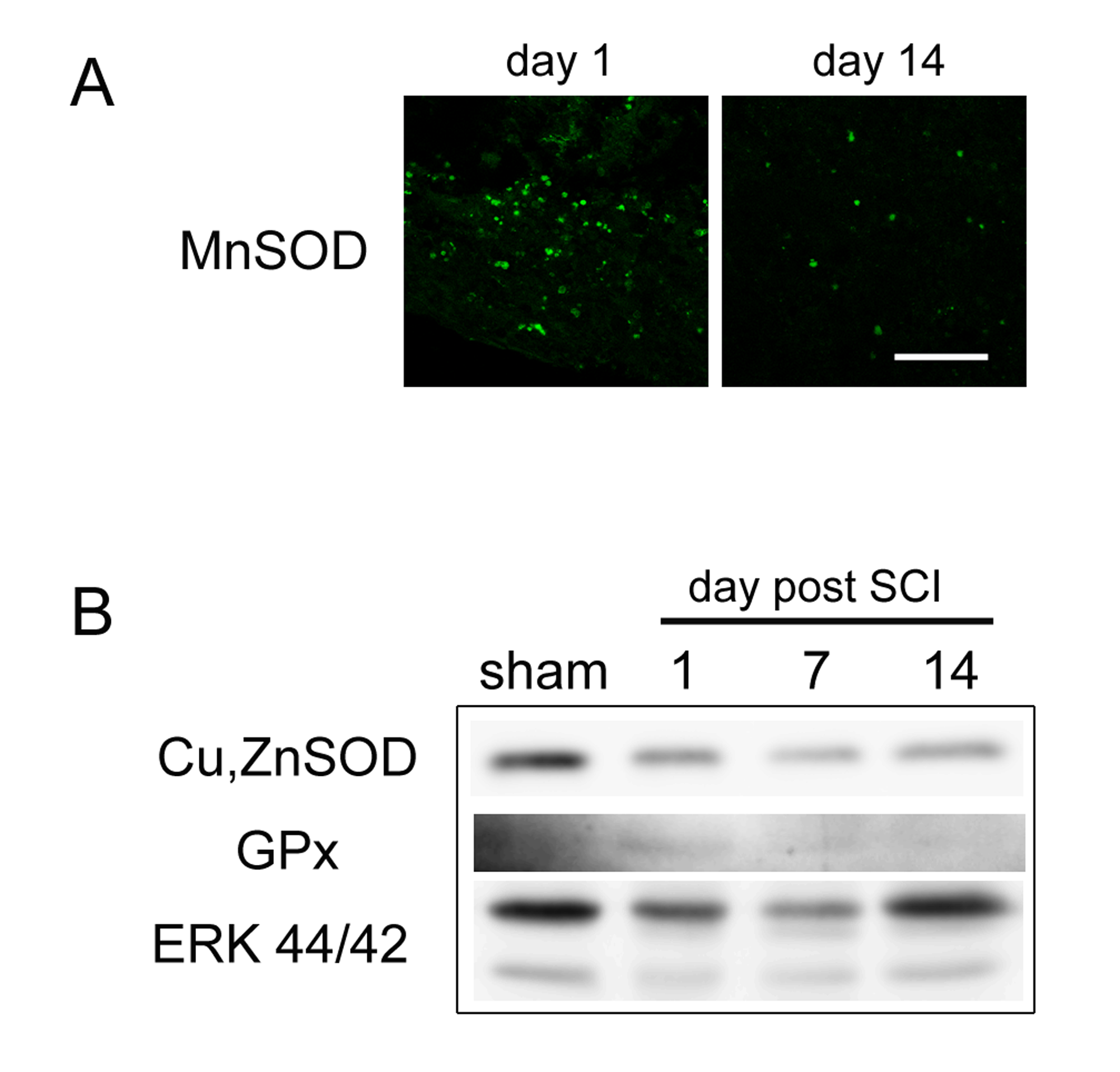

Supplement: Additional file 1 — Figure S1: Examination of MnSOD, Cu,ZnSOD and GPx expression in the lesion center at day 1 and day 14 post SCI. (A) The injured spinal cord tissue sections were collected at day 1 and day 14 (E,F) after SCI, and then subjected to immunofluorescence for MnSOD. There were numerous MnSOD+ cells observed in the lesion center at day 1 post SCI, while few MnSOD+ cells were found in the lesion center at day 14. Scale bar, 50 μm. (B) Western blot analysis showed the reduction of Cu,ZnSOD and GPx levels at the lesion center when compared to that detected in the sham control. The proteins extracted from the lesion center of the injured spinal cords at the different survival time points (day 1, 7 and 14) after SCI or from sham control. The same blot was stripped and reprobed with ant-ERK44/42 antibody as internal loading control. [file 1423-0127-18-13-S1.TIFF]

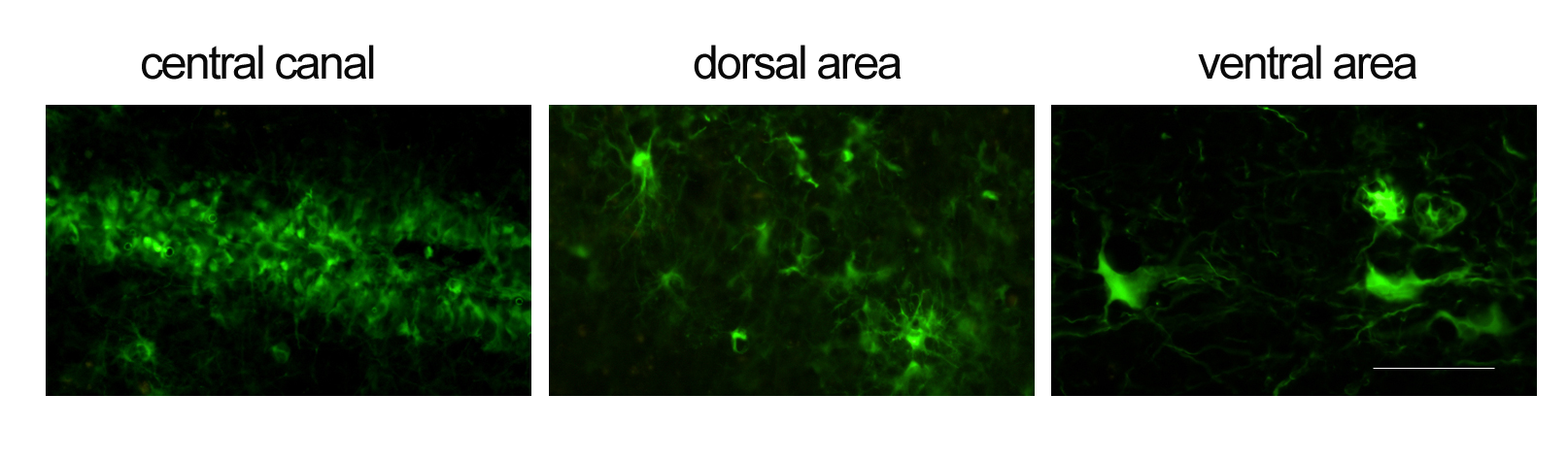

Supplement: Additional file 2 — Figure S2: Green fluorescent protein (GFP) expression in ependymal cells lining along the central canal of the spinal cord and in neural cells located at the dorsal and ventral portions of the injured spinal cord. rAd-GFP (1 × 10^8^ pfu/injection) was injected into the rat spinal cord 1 mm rostral to the lesion center within 10 minutes after SCI. The rats were sacrificed at 7 days post SCI, and perfused in 4% paraformaldehyde. The injured spinal cord tissues were removed and prepared for cryostat as described in Experimental Section. Scale bar, 25 μm. [file 1423-0127-18-13-S2.TIFF]
